# Interdisciplinary Management of Bilateral Palatally Impacted Canines With Mini-Screws as Temporary Anchorage Devices: A Case Report

**DOI:** 10.7759/cureus.67978

**Published:** 2024-08-27

**Authors:** Joann Sook Mei Yong, Umi Mardhiyyah Mat Ali

**Affiliations:** 1 Orthodontic Unit, Prestige Dental & Oral Facial Surgery Specialist Clinic, Selangor, MYS; 2 Orthodontic Unit, School of Dental Sciences, Universiti Sains Malaysia, Kelantan, MYS

**Keywords:** fixed appliance, interdisciplinary treatment approach, temporary anchorage devices, mini-screws, palatally impacted canines

## Abstract

Dealing with impacted maxillary teeth can be quite challenging for dental professionals. The advantage of using temporary anchorage devices (TADs) in the treatment of bilateral palatally impacted canines is the ability to exert more controlled and directed forces on the impacted tooth. Utilizing TADs might result in enhanced outcomes, including better tooth positioning within the dental arch and reduced risk of complications, such as root resorption. This article highlights the importance of diagnosis, adequate treatment planning for the eruption of impacted canines, and also managing tooth-arch size discrepancy to achieve a balanced occlusion and dental aesthetic. This is a case report on the interdisciplinary management of bilateral palatally impacted canines using mini-screws as TADs.

## Introduction

The impacted canine has been recognized as a complex and demanding problem within the field of orthodontics, particularly when the canines are impacted and displaced in a palatal position. Addressing palatally impacted canines (PICs) has involved various orthodontic techniques and surgical interventions. This problem necessitates the collaboration between the oral maxillofacial surgeon and the orthodontist, first to gain access to the impacted teeth and subsequently to employ precise orthodontic biomechanics to position the tooth correctly. Traditionally, orthodontic treatment has relied heavily on the use of braces, brackets, and wires to guide the movement of teeth [1]. This approach, while effective, often requires considerable time and effort to achieve the desired outcomes. However, recent advancements in orthodontics have paved the way for more efficient and innovative tooth movement strategies [2]. One such approach is the utilization of mini-screws as temporary anchorage devices (TADs), which have gained significant recognition in the field of orthodontics. This small device provides skeletal anchorage, allowing for more targeted tooth movement control without relying solely on adjacent teeth. It also offers increased precision, reduced treatment times, and improved patient comfort [3]. This case report describes the interdisciplinary management of bilateral PICs using mini-screws as TADs.

## Case presentation

Case history

**Figure 1:**
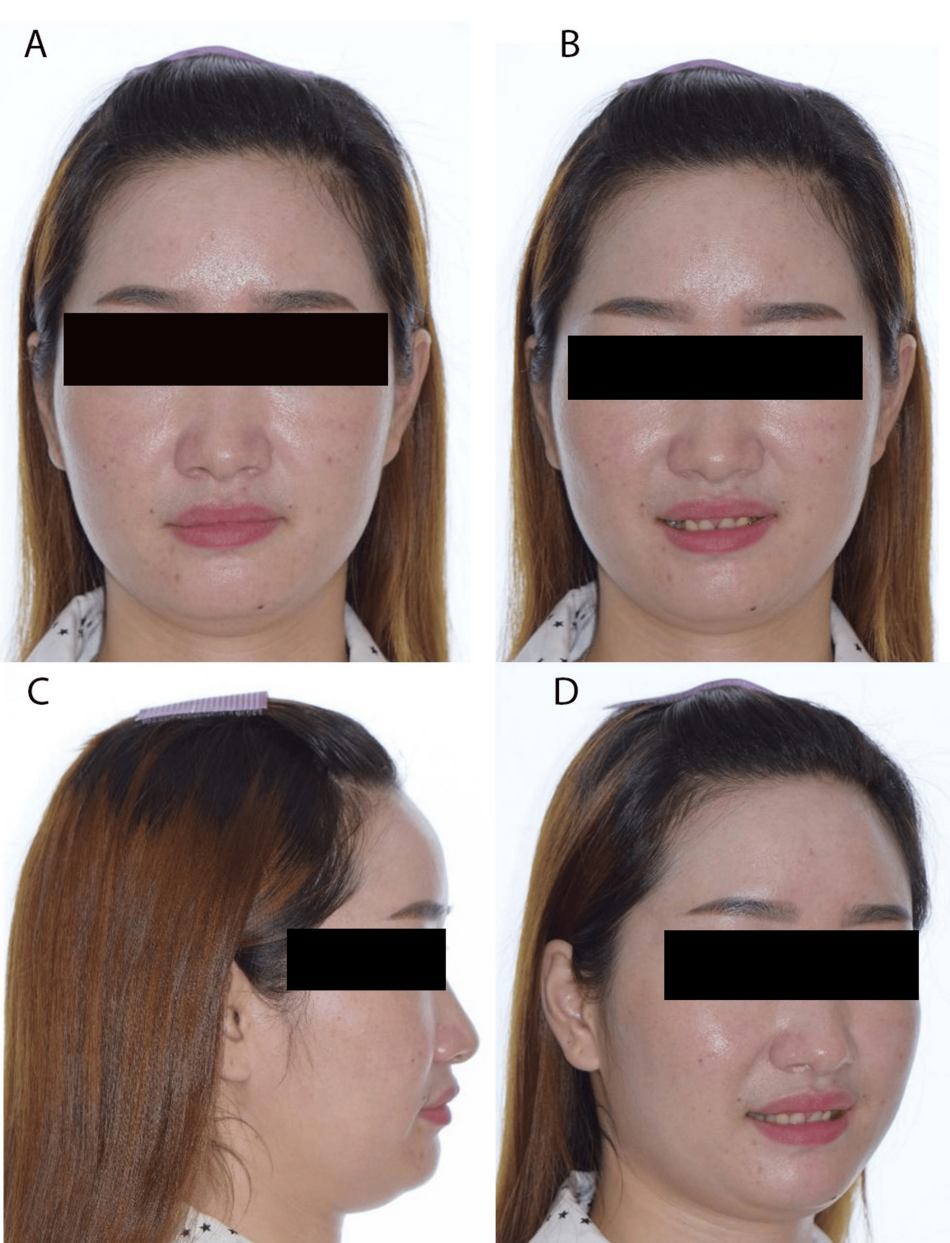
Pre-treatment extra-oral photographs A: Frontal rest position; B: Frontal smile position; C: Lateral position; D: 45-degree position

A medically fit and healthy 33-year-old Chinese female was concerned about her loose upper deciduous canines. She was presented with a Class I skeletal pattern, reduced Frankfort mandibular plane angle, and reduced lower anterior face height. Her chin point was slightly deviated to the left. Soft tissue examinations revealed competent lips, average nasolabial and deep labiomental angles and a retrusive profile in relation to the Ricketts E-line (Figure 1). The upper and lower dental midline was coincidental with the facial midline. There was a flat smile arc with reduced teeth during smiling, and a high lower lip line at rest. No signs and symptoms of temporomandibular dysfunction joint were present.

**Figure 2:**
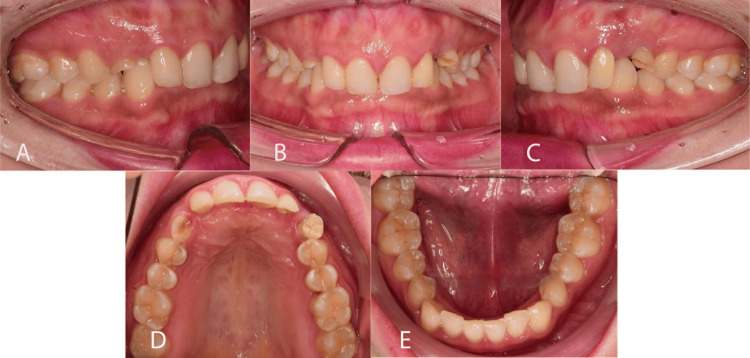
Pre-treatment intra-oral photographs A: Right lateral view; B: Frontal view; C: Left lateral view; D: Upper occlusal view; E: Lower occlusal view

Intra-oral examination (Figure 2) revealed a full permanent dentition except 18, 28, 13, and 23. There were retained upper deciduous canines bilaterally that were attrited and mobile. Overall, there was generalized microdontia. A healing sinus was noted on the labial aspect of 22. Root canal treatment of 22 was completed before the orthodontic examination.

The maxillary arch form was U-shaped with spacing distal to 12 and 22 and retained both upper deciduous teeth. The upper incisors appeared retroclined and supraerupted, and both the upper lateral incisors had composite crowns. There was a palpable bulge palatally on both sides. The mandibular arch form was U-shaped with mild crowding, average incisor inclination and deep curve of Spee.

In occlusion, the incisor relationship was Class II Division 2 with an overjet of 2mm. The overbite was increased 100% and complete to the palate but atraumatic. The upper and lower center lines were coincidental. The buccal segment relationship was Class I bilaterally. 

Radiographic examination

**Figure 3:**
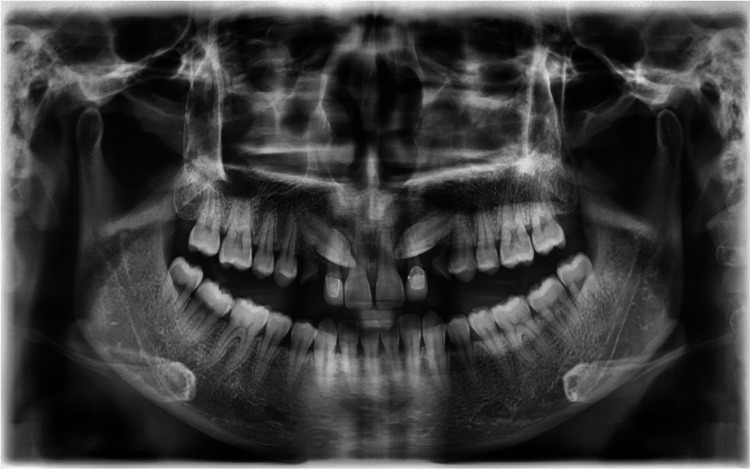
Pre-treatment orthopantomogram

A panoramic examination (Figure 3) revealed that all teeth were present except teeth 18 and 28. Teeth 13 and 23 were mesially angulated, and its follicle appeared normal. The apex of the permanent upper canines was fully formed and situated above the adjacent upper first premolars. The crown tip of 13 has crossed the distal aspect of 11. Vertically, it was at the cervical third of the crown of 11. However, the crown tip of 23 was at the distal aspect of 21, and vertically, it lay at the mid root of 21. Given the patient’s age, the prognosis for alignment of 13 and 23 was fair. Bone levels were good, and no abnormal pathology was detected.

CBCT assessment

**Figure 4:**
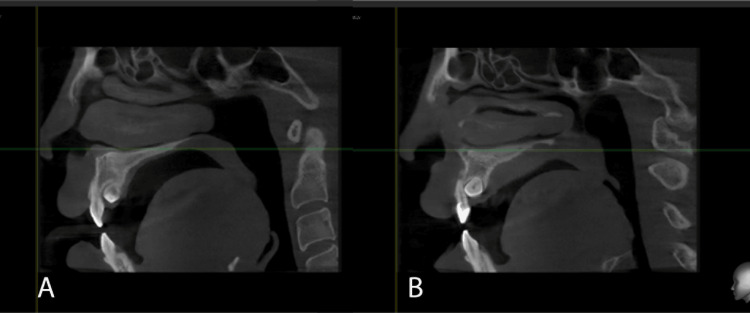
Pre-treatment CBCT CBCT: Cone-beam computed tomography A: Tooth 13; B: Tooth 23

CBCT (Figure 4) was taken to confirm the palatal position of 13 and 23. The roots of 12 and 22 were seen out of the bony envelope. There was no evidence of follicular enlargement or root resorptions to adjacent teeth on both sides of the impacted teeth. The index of treatment difficulty score according to 3D CBCT guide is 8; thus, the best treatment intervention for this case is surgical exposure and orthodontic traction [4].

Treatment objectives

The treatment aims were to disimpact bilateral PICs and achieve good tooth alignment, close the spaces, improve the overbite, improve soft tissue balance, improve smile aesthetics, and establish stable functional occlusion.

Treatment plan and alternatives

The patient was informed about several treatment options for the bilateral PICs: (i) Surgically exposed and orthodontic traction; (ii) Surgical removal and replacement with implants/ bridge/ denture; (iii) Do nothing and monitor with bridge/denture replacement

She chose the first option due to the additional cost burden of implants and other potential risks involved. As the correction of facial asymmetry was not her main priority, she chose to accept her condition and focused on the dental correction.

Treatment progress

**Figure 5:**
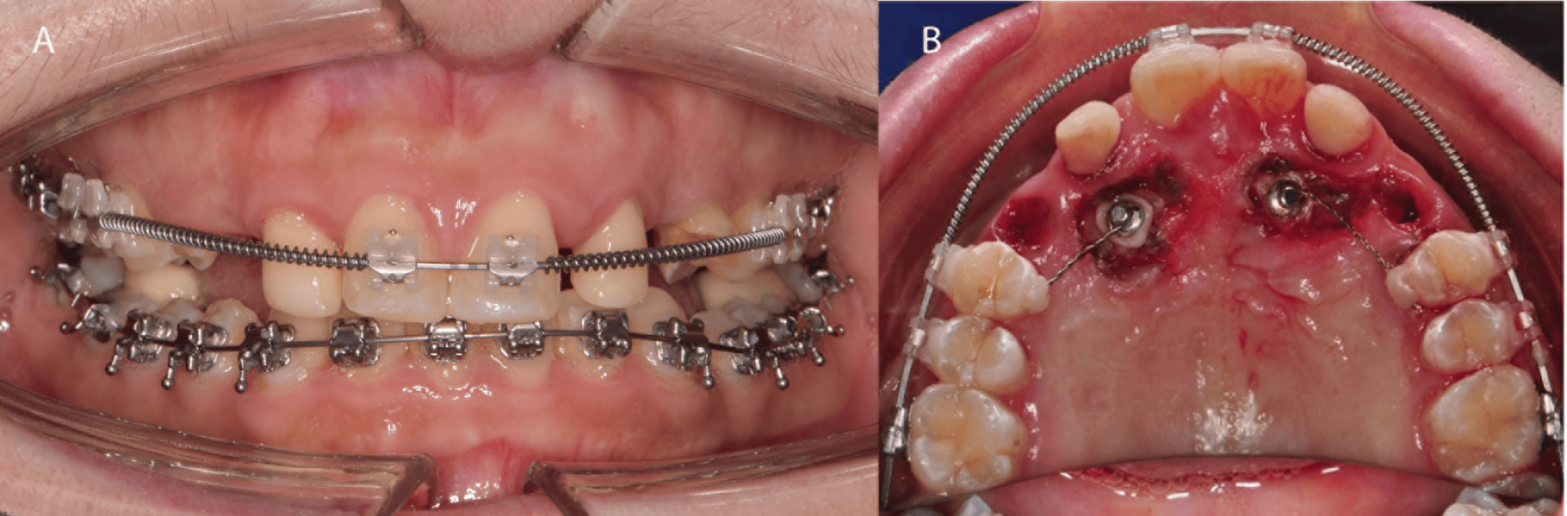
Space creation and surgical exposure A: Space creation using Niti open coil spring; B: Surgical exposure of 13 and 23

Treatment was initiated with alignment of the upper arch using self-ligating fixed appliance (MBT prescription), 0.022”x0.028'' archwire slot (TOMY Inc, Japan) bypassing teeth 12 and 22, and the upper deciduous canines, using 0.012” Nickel Titanium (Ni-Ti) archwire. After initial alignment, space creation for the upper arch was made by Ni-Ti open coil spring between 11 and 13 and 21 and 23 using 0.016” Ni-Ti (Figure 5A), and at the same time, the lower arch was bonded with 0.014” Ni-Ti. The leveling and alignment phase were continued on the upper arch with 0.018”x0.025” Ni-Ti, 0.019”x0.025” stainless steel archwire before the patient was referred to the oral maxillofacial surgeon for surgical exposure of 13 and 23 (Figure 5B).

**Figure 6:**
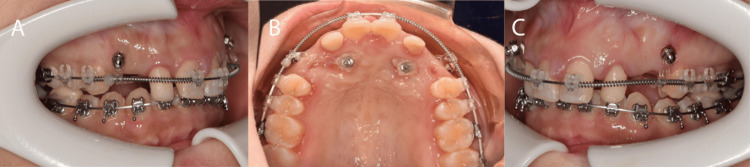
Placement of mini-screws to disimpact 13 and 23 A: Right lateral view; B: Upper occlusal view; C: Left lateral view

Open exposure was done, and a stainlesssteel button with ligature ties was bonded on the labial surface of 13 and 23. The orthodontic traction commenced on the next visit with the insertion of two mini-screws size 1.6mm x 8.0mm, stainless steel (BOMEi Co., Ltd, Taiwan) on the buccal bone mesial to the upper first premolars (Figure 6).

In the meantime, the lower arch was leveled with the following sequence: 0.018” Ni-Ti and 0.017” x 0.025” Ni-Ti until the final working archwire 0.019” x 0.025” stainless steel. After six months, overbite correction began by adding a reverse curve of Spee on the lower archwire.

As the orthodontic traction continued on the upper canines, 12 and 22 slowly began to extrude. During traction, the direction of the vector to the upper canines was also changed to a more distal vector.

**Figure 7:**
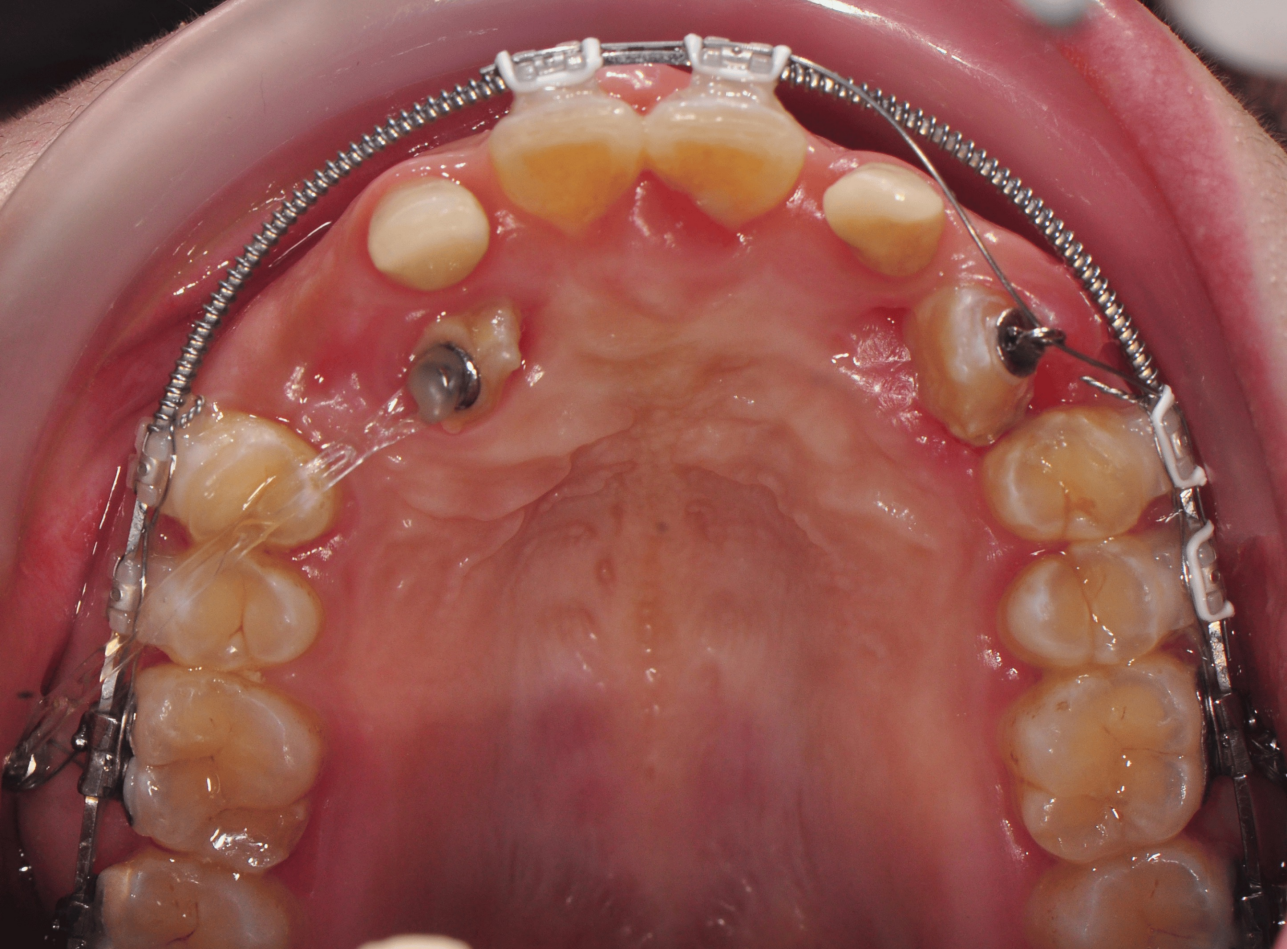
Piggyback method with base archwire of 0.019” x 0.025” stainless steel and 0.012” Ni-Ti

After four months of orthodontic traction, 23 began to erupt, and its eruption was assisted using the piggyback method with 0.012” Ni-Ti (Figure 7).

By the fifth month of orthodontic traction, the tip of the crown of 13 could be seen. Both upper canines were aligned using the same piggyback method until the crown of the canines fully erupted. Then, a continuous archwire was inserted, and the upper lateral incisors were bonded.

The upper arch was realigned again until the final working archwire of 0.019” x 0.025” stainless steel was reached. Space closure was commenced with Class I traction on the upper arch and Class II elastic 3.5oz (1/4”) (ORMCO, CA). In the meantime, spaces were redistributed for the build-up of the upper lateral incisors.

At the finishing stage, the root of the 22 was uprighted with a second-order bend for root parallelism before the tooth was built up using a 0.018” stainless steel archwire.

**Figure 8:**
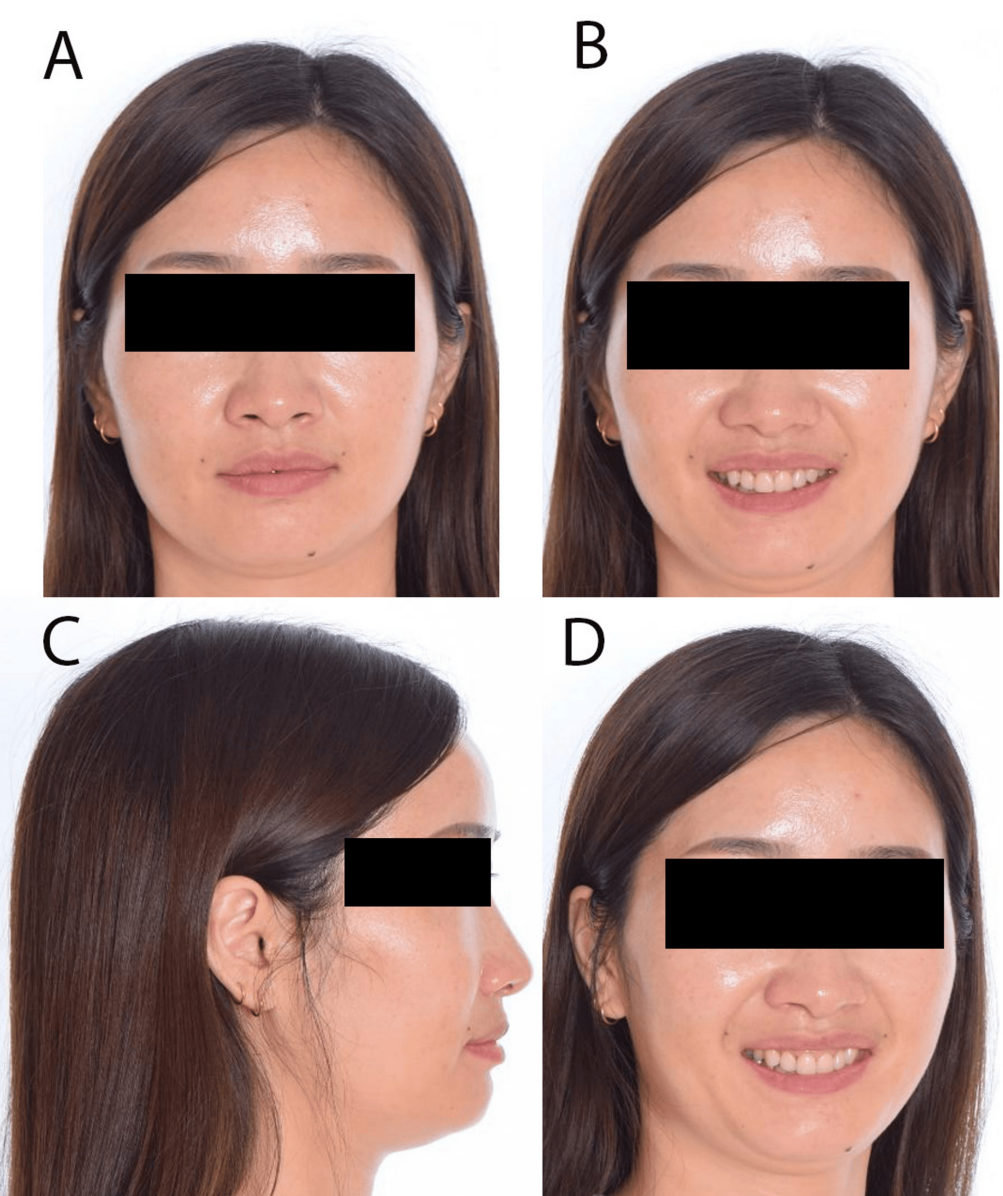
Post-treatment extra-oral photographs A: Frontal rest position; B: Frontal smile position; C: Lateral position; D: 45-degree position

**Figure 9:**
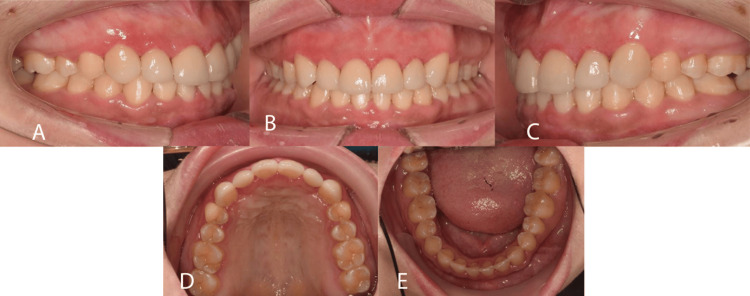
Post-treatment intra-oral photographs A: Right lateral view; B: Frontal view; C: Left lateral view; D: Upper occlusal view; E: Lower occlusal view

**Figure 10:**
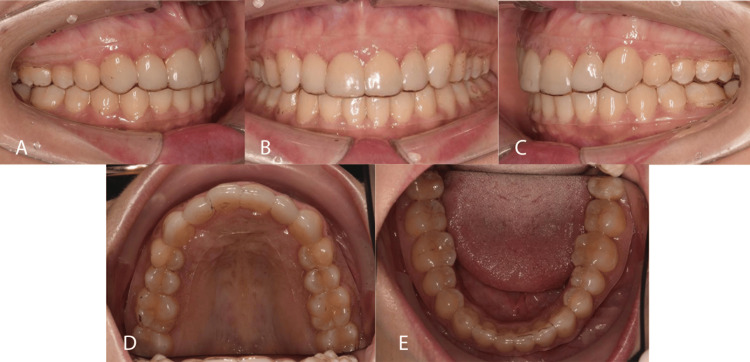
One-year post-treatment with vacuum-formed retainers A: Right lateral view; B: Frontal view; C: Left lateral view; D: Upper occlusal view; E: Lower occlusal view

Before debonding, the patient was referred to the general dental practitioner to discuss the build-up choices, and she decided to go with porcelain veneers for the first six anterior teeth. All appliances were removed after 27 months of active treatment. Upper and lower vacuum-formed retainers were delivered. The patient was instructed to wear the retainers full-time for the first six months and nighttime thereafter (Figures 8-10). The pre-and post-treatment cephalometric changes are shown in Table 1. The major changes can be seen in the dental components, where the upper and lower incisors are more proclined post-treatment.

**Table 1:** Pre and Post-treatment lateral cephalometric values by Eastman analysis SNA: Sella-Nasion to A point angle; SNB: Sella-Nasion to B point angle; ANB: A to B point angle; MMPA: Maxillo-mandibular plane angle; LAFH: Lower anterior face height; SN: Sella-Nasion; APog: A point to Pogonion

Variables	Mean	Pre-treatment	Post-treatment	Difference
SNA	81°±3	82.1°	81.8°	0.3°
SNB	78°±3	78.0°	78.5°	0.5°
ANB	3°±2	4.1°	3.3°	0.8°
MMPA	27°±5	18.0°	18.5°	0.5°
LAFH ratio	55±2%	52%	52.5%	0.5%
SN to maxillary plane	8°±3	11°	11°	0°
Upper incisor to maxillary plane	109°±6	91.5°	112.7°	21.2°
Lower incisor to mandibular plane	93°±6	91.2°	98.5°	7.3°
Interincisal angle	135°±10	159.3°	132.8°	26.5°
Wits appraisal (mm)	0±1	-1.6	-2.3	0.7
Lower incisor to APo line (mm)	1± 2	-2.4	0.9	1.5

## Discussion

The present case report highlights the interdisciplinary approach to managing bilateral PICs. This approach involved a combination of orthodontic treatment, surgical exposure, mini screws for anchorage, and restorative finishing after orthodontic treatment. The first step in the treatment was bonding the patient's teeth and bypassing the adjacent teeth 12 and 22. Utilizing the "free body" technique safeguards neighboring teeth while relocating the impacted tooth within the dental arch. Without a bracket, the upper lateral incisors will be able to move away from the pressure a tooth follicle exerts, thus preventing root resorption [5].

A radiographic image of the location of impacted upper canines is essential for making a treatment plan because it shows the accessibility of the tooth for surgery and where to apply the orthodontic forces [6]. CBCT, as a radiographic modality, provided detailed information about the location of impacted canines and root resorption on adjacent teeth. In this case, the CBCT revealed that both PICs were in close proximity to the upper lateral incisors' roots. However, no root resorption was noted. Careful evaluation of the radiographic images can help predict the difficulty of the surgical exposure and the ensuing orthodontic traction and guide the most appropriate treatment approach [7].

The first reason for creating adequate space orthodontically is to take advantage of the regional acceleratory phenomenon effect immediately after surgical exposure to tract the PIC. The osteogenic activity is usually high after surgery due to the migration of inflammatory factors. Therefore, the speed of orthodontic traction and tooth movement are greatly enhanced [8-10]. By leveraging the regional acceleratory phenomenon, remarkable tooth movement and positional changes can be achieved in a significantly reduced timeframe compared to traditional orthodontic approaches [11].

The decision was made to perform open exposure on the canines. The technique was selected based on the orthodontist’s preference and the most optimal option for this case [12]. Uncovering the palatal impacted canines allowed exact orthodontic force vectors to be visualized and applied directly to the teeth. 

Mini-screws offered a fixed and stable anchorage point for orthodontic mechanics to bring impacted canines into the dental arch. This innovative technique utilizes small implanted screws placed in the buccal cortical bone to serve as the anchorage, allowing direct control of the impacted canine without relying on other teeth for support [1]. In this case, the mini-screws were placed at the buccal area mesial to the upper premolars to tract down the crown before changing to an infra zygomatic crest area between the first and second upper permanent molars. The changes in the location of the mini-screws were also done to change the direction of force to be more distally.

Once the PICs were visible, the piggyback technique was used to align it further. The piggyback technique involved placing a flexible archwire on top of the existing rigid or passive archwire to continue guiding the impacted tooth into its final position. After achieving alignment of the PICs, further orthodontic treatment was carried out to ensure proper occlusion and alignment of the surrounding teeth. This technique was considered more cost-effective and removed the issues with the activation and deactivation pressures produced along a continuous archwire [13].

Post-treatment, ideal Class I occlusion was achieved bilaterally with a functionally stable cusp-embrasure relationship. Nevertheless, a significant deep bite of around 50% persisted, potentially because of the extended length of the veneer. Additional palatal root torque should be applied to the upper left canine to ensure optimal final positioning. However, the patient expressed satisfaction with the outcomes. The outcomes obtained remained stable one year after the treatment.

## Conclusions

This case report demonstrates the effectiveness of the interdisciplinary approach and mini-screws' utilization in producing excellent outcomes in complicated orthodontic cases with bilateral PICs. Utilizing mini-screws as TADs has significantly expanded the range of orthodontic treatment options, allowing for more complex and precise tooth movements that were previously challenging or even impossible with conventional techniques.
